# Recurrence of Dupuytren’s contracture: A consensus-based definition

**DOI:** 10.1371/journal.pone.0164849

**Published:** 2017-05-15

**Authors:** Hester J. Kan, Frank W. Verrijp, Steven E. R. Hovius, Christianne A. van Nieuwenhoven, Dupuytren Delphi Group, Ruud W. Selles

**Affiliations:** 1 Department of Plastic and Reconstructive Surgery and Hand Surgery, Erasmus Medical Center, Rotterdam, the Netherlands; 2 Department of Rehabilitation Medicine and Physiotherapy, Erasmus Medical Center, Rotterdam, the Netherlands; Nanjing Medical University, CHINA

## Abstract

**Purpose:**

One of the major determinants of Dupyutren disease (DD) treatment efficacy is recurrence of the contracture. Unfortunately, lack of agreement in the literature on what constitutes recurrence makes it nearly impossible to compare the multiple treatments alternatives available today. The aim of this study is to bring an unbiased pool of experts to agree upon what would be considered a recurrence of DD after treatment; and from that consensus establish a much-needed definition for DD recurrence.

**Methods:**

To reach an expert consensus on the definition of recurrence we used the Delphi method and invited 43 Dupuytren’s research and treatment experts from 10 countries to participate by answering a series of questionnaire rounds. After each round the answers were analyzed and the experts received a feedback report with another questionnaire round to further hone in of the definition. We defined consensus when at least 70% of the experts agreed on a topic.

**Results:**

Twenty-one experts agreed to participate in this study. After four consensus rounds, we agreed that DD recurrence should be defined as “more than 20 degrees of contracture recurrence in any treated joint at one year post-treatment compared to six weeks post-treatment”. In addition, “recurrence should be reported individually for every treated joint” and afterwards measurements should be repeated and reported yearly.

**Conclusion:**

This study provides the most comprehensive to date definition of what should be considered recurrence of DD. These standardized criteria should allow us to better evaluate the many treatment alternatives.

## Introduction

Recurrence of disease following any technique to correct the contracture(s) is one of the major setbacks in the treatment of Dupuytren’s disease (DD). Since present techniques only treat the symptoms of this chronic and progressive disease, recurrence over time is inevitable in the majority of patients. Therefore, assessment of recurrence rates is an essential element in describing and comparing the efficacy of different treatment options for DD.

Two separate systematic reviews [1, 2] have recently identified dire need for consensus on how to define recurrence of DD. This lack of a clear definition may partly explain why reported recurrence rates vary from 0% to 100% [3–8]. In addition, we have shown that applying the different definitions on a single dataset can change the resulting recurrence rates from 2% to 86%[1].

To obtain an internationally accepted and wide supported definition of recurrence for DD, a consensus agreement based on the experience and knowledge of an international group of renowned experts is needed. Therefore, the goal of this international study was to develop consensus on a single definition of recurrence of DD that is applicable in clinical and research settings.

## Methods

In this study we used the Delphi method, which is designed to reach consensus between individuals using questionnaire-based surveys [9]. This expert-based consensus study did not involve participation of study subjects such as patients or non-patient volunteers. Therefore, no institutional review board approval was needed for the present study based on local law. Experts in the field of Dupuytren’s disease (DD) were invited to participate in our Delphi study. To identify these experts, we selected all clinical DD-related PubMed articles that were published between 2005 and 2012. In addition, we used the articles from our systematic review to identify experts in the field of DD [1]. Either the first or last author of each article, based on the number of publications in the field of DD, was invited to participate. When multiple experts were identified from the same institution, only the most experienced expert was invited to participate. We excluded experts that did no longer participate in the field, for example due to retirement, or authors who published only a single DD-related paper.

In November 2012, 42 experts from ten countries in four continents were invited to participate. All experts were provided with information on the Delphi study as well as with a draft of our systematic review. Following Delphi guidelines, 51% agreement is considered consensus. However, we aimed for a minimal of 70% agreement for consensus. The identities of the other participating experts were not disclosed to the experts during the process.

In the first round, experts were asked to score the relevance of four different dimensions of recurrence to be included in a single definition of DD recurrence (first two columns of Table 1) using a 0–10 numerical scale and multiple choice questions. For example, we asked “On a scale from 0 to 10, how important is it to include the return of Dupuytren’s nodules based on palpation or visual inspection in the definition of recurrence?” After each question, the experts could add a comment or explanation.

**Table 1:** Dimensions. The dimensions (numbered 1–4) were presented to the experts and the resulting consensus on each dimension is presented. The last column shows the percentage of experts that agreed on each consensus or a range of percentages, when the outcome differed in more than one round of the Delphi study.

	Dimensions	Consensus	% Experts
1	Location of recurrence	All treated joints	70%–80%
2	Inclusion of nodules, cords and contractures	20° contracture, no modules or cords	86%
			56%–60%
3	Baseline measurements and follow-up	6 weeks post treatment, 1 year post treatment	79%
			86%
4	Patient characteristics & Patient-reported recurrence	Excluded	75%

The first two authors analyzed the results and discussed the outcomes with the other authors. If 70% of the experts scored five or higher, the item was considered important for further consideration. These included items were discussed more in-depth in the following rounds.

In each following round, we provided feedback to the experts by summarizing the answers on the previous round in combination with a synopsis of anonymous comments. After this feedback, we asked the experts to answer each question again on which consensus was not yet reached. Topics on which consensus was reached were also presented but only with the opportunity for the experts to give additional comments. If experts did not complete a previous round before the deadline, they were still invited to the next round.

## Results

**Fig 1:**
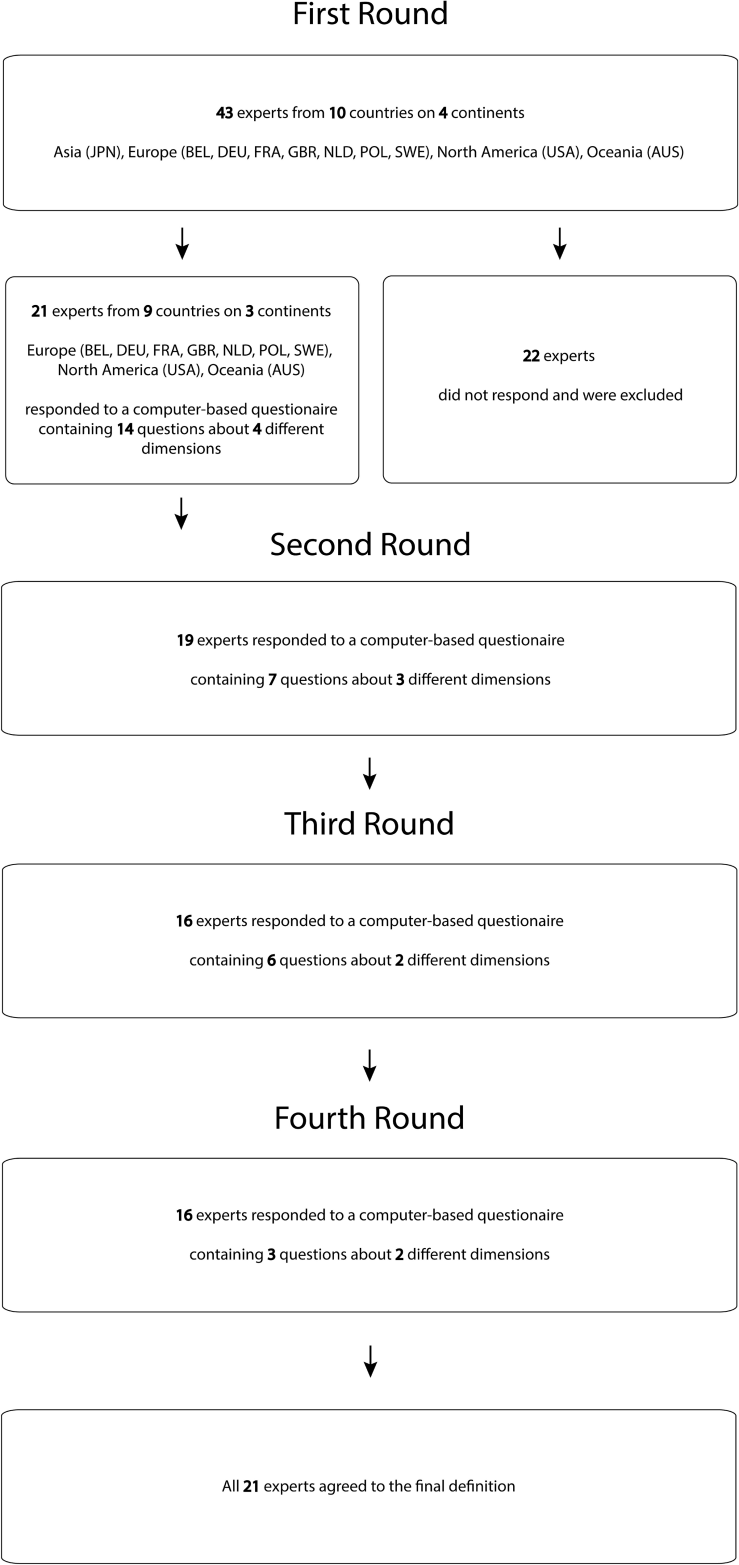
Flow diagram. Figure showing shows the number of experts who were included in the study rounds and their country of origin.

Twenty-one experts (64%) from 10 countries participated in this study: 7 from North-America, 13 from Europe, and 1 from Australia. A total of four rounds were needed to reach consensus. The response rate varied per round between the 76% and 90% (Fig 1).

A first dimension scored by the experts was location of recurrence. Consensus was that recurrence of Dupuytren’s disease (DD) should be located in the operated area only in order to differentiate recurrence from disease extension to other joints. In addition, since DD can affect multiple joints, fingers and hands, consensus was that recurrence should be measured in all treated joints, fingers and hands regardless if full extension was reached during treatment. Experts also reached consensus that all treated joints should be scored individually to count as a recurrence rate (Table 1).

The second dimension was whether a recurrence should be assessed based on the presence of nodules, cords and/or joint contractures. Experts agreed DD nodules and cords should not be explicitly taken into account and furthermore a recurrent joint contracture of at least 20 degrees in one joint is needed for a recurrence.

A third dimension was the timing of baseline measurements and follow-up. Experts agreed recurrence should be measured at one year post-treatment and should be compared to a baseline measurement. Consensus was that intra-operative measurements should not be used as a baseline value and, therefore, an assessment at six weeks after treatment was selected as a baseline. Since it is presently unclear from literature how recurrence develops over time, experts agreed to recommend yearly repeated measurements when feasible.

A fourth dimension consisted of scoring patients’ characteristics, such as diathesis and patient perception of recurrence. Although it is clear that diathesis has a significant influence on recurrence, the experts agreed that information on diathesis should not be included into the definition, although it should be scored in every study. The experts also agreed that, while patient-rated information about recurrence can be relevant, it should not be included in a single definition of recurrence of DD.

After the last round, all 21 experts agreed to define recurrence of Dupuytren’s disease after treatment as *“an increase in joint contracture in any treated joint of at least 20 degrees at one year post-treatment compared to six weeks post-treatment”*. Additionally, although not part of the definition, the experts advised the community to 1) conduct studies that repeat measurements yearly to study the development of recurrence, and 2) measure and report recurrence rates for all treated joints individually (Table 2: implementation of the definition).

**Table 2:** Examples of recurrences. Table showing a fictitious cohort of Dupuytren’s patients and shows when recurrence has occurred by using the consensus definition. It also shows the recurrence rate that should be described in the paper.

Patient	Hand	Joint	Extension deficit prior treatment (degrees)	Extension deficit 6 weeks post treatment (degrees)	Extension deficit 1 year post treatment (degrees)	Recurrence (Yes / No)	Recurrence rate (%)
1	Left	MP 4	60	10	10	No	5/14 joints = 36%
		MP 5	75	0	20	Yes	
	Right	MP5	20	0	0	No	
		PIP 5	90	40	60	Yes	
2	Left	MP 5	30	10	15	No	
		PIP 5	80	20	35	No	
3	Right	MP 4	10	0	10	No	
		MP 5	15	0	15	No	
		PIP 5	40	0	20	Yes	
4	Left	PIP 5	90	10	25	No	
5	Left	MP 3	60	10	30	Yes	
		MP 4	40	0	15	No	
		MP 5	30	0	15	No	
		PIP 5	60	5	25	Yes	

## Discussion

Since the present lack of a consensus for recurrence of Dupuytren’s Disease make it impossible to compare results between different studies, we conducted this international study to obtain consensus on a universal definition for recurrence of DD after treatment. Based on this, we propose to define recurrence of DD after treatment as *“an increase in joint contracture in any treated joint of at least 20 degrees at one year post-treatment compared to six weeks post-treatment*

The definition established in this study was obtained by evaluation four different dimensions of recurrence. The first dimension was location of recurrence. Consensus was that only the operated or treated area should be considered and that all treated hands, fingers and joints should be included to calculate recurrence rates, which allow to distinguish recurrence (in the same area) from disease extension (outside of the treated area). In addition, although additional measures such as a total passive extension deficit (TPED) can also be of value, consensus was that individual joint measurements should be used primarily. One expert stated: ‘TPED is measured while all joints are being simultaneously passively extended. As such, it represents fixed joint contractures. This will yield a different measurement than the sum of measurements made of individual joint passive extension, while the proximal joint or distal joints in that same ray are allowed to flex.’ Furthermore, a disadvantage of a TPED is that it includes non-affected joints and newly affected joints (disease extension), creating possible false-positive recurrence rates.

A second dimension considered including palpable nodules, palpable cords and contractures in the definition of recurrence. The experts unanimously agreed to include increase of contracture in the definition of recurrence. Furthermore, they agreed to exclude nodules and cords. The angular threshold for the contracture to be considered a recurrence was set at 20 degrees. There were two reasons for this threshold. Firstly, inherent measurement errors of goniometry are approximately 5–10 degrees and therefore a larger threshold is needed [10]. Secondly, 15–20 degrees is often considered an indication for a new intervention, for example in the Hueston Table-top test [11].

The exclusion of the presence of nodules and cords in the definition was more controversial in our group of experts. While the main reason to include palpable nodules and palpable cords in the definition was that reappearing nodules and cords are the earliest signs and often the cause of recurrence, the majority of the experts mentioned three main reasons to exclude palpable nodules and palpable cords in the definition. Firstly, nodules and cords by themselves very seldom cause any disability, or require surgical treatment. Secondly, minimal invasive techniques are meant to disconnect Dupuytren tissue that forms cords or nodules. However, these cords and nodules are left in place during these techniques [5, 12]. This makes it difficult to identify newly formed nodules and cords because the old ones remain. Thirdly, it is challenging to reliably identify the presence of nodules and cords in the presence of post-surgical scarring.

A third dimension considered timing of baseline and follow-up measurements. Consensus was to perform baseline measurements at six weeks post treatment, mainly because experts concluded that wound healing takes time following surgery. Furthermore, hand function will return in approximately two to four weeks and it also has been demonstrated that the results at six weeks post treatment were better compared to one-week post treatment [13, 14]. Therefore, six weeks was considered a first time-point evaluation for treatment success. The follow-up time was more controversial. Experts mentioned from a clinical point of view, longer follow-up measurements might express more precisely the amount of recurrent treatments that are needed. However, from a research perspective, a one-year follow-up may already express the main differences between techniques. One expert stated: ‘recurrence progresses with time. But this progression is non-linear. Either our scientific community develops standardized time-to-recurrence charts, or we all decide to evaluate all patients at a given point in time.’ After four rounds, consensus was to measure recurrence after one year. In addition, the experts advised yearly repetition of measurements in studies that cover multiple follow-up years since more knowledge is needed on how recurrence progresses over time.

A last dimension included patient characteristics and patients’ perception. Consensus was that patient factors (e.g. diathesis) can predict the risk of developing recurrence, but are not a characteristic of recurrence itself [15]. Therefore, it was excluded. In addition, while all experts concluded that patients’ perception is very important [16], it was also excluded. One experts stated ‘while we can pat ourselves on the back for a great range of motion improvement, or feel we did not achieve our goal, the patient's own perception is the bottom line of what matters the most. Unfortunately, we do not have very objective measures (of subjective improvement) and any measure will be invariably affected by factors unrelated to the medical treatment delivered’. Since, there are no objective measures to measure patients’ perception about recurrence, it is not included in this definition.

Our study has a number of weaknesses and strengths. Firstly, only the minimal amount of experts generally assumed to be needed for a Delphi study participated in our study [9]. Unfortunately the invited experts from the Asian continent did not respond and are therefore not represented in this Delphi study. However, all responded experts represent countries from all over the world and are clearly renowned in the field. Experts completed all rounds with an average response rate of 80% and, at the end of the process, all experts agreed on the final definition of recurrence. Secondly, this Delphi study was conducted with computer-based questionnaires. A disadvantage of this method is that it lacks the ability to stimulate discussion and can lead to misinterpretation of comments given by experts. On the other hand, computer-based questionnaires allow anonymous responses from the experts, and thus avoiding possible peer-pressure. A third limitation was that the goniometric measurement protocol needed for this definition was not part of the Delphi consensus rounds. To our knowledge, an internationally recognized guideline for measuring joint angle is presently lacking. In our experience, most researchers and clinicians measure joint angle dorsally [17]. As some of the experts as well as a reviewer of this manuscript have correctly noted, it is important to control for the adjacent joints when measuring a specific joint, especially when a cord spans multiple joints. Fortunately, since the present definition is based on a change in joint angle of time, differences between goniometric measurement techniques may lead to different absolute angles, but difference may be much smaller when analyzing the change in joint angle over time. A final limitation is that while our goal was to obtain one clinically relevant and easily applicable definition for recurrence of DD after treatment, it may not be possible to reflect the complexity of recurrence of DD in this single definition. Table 2 shows an example of how a typical dataset from a clinical study should be interpreted to calculate a recurrence rate. From this table, it is also clear that this single recurrence rate does not capture the complexity of the data. Therefore, we do not advocate researchers to only use this single measure, but we do advocate this is the minimal measure to report. Additional secondary measures may be needed to also describe the presence of the disease or disease extension, for example the presence of palpable nodules and cords. Also, in addition to using a threshold for recurrence, it could also be valuable to describe the average change in joint angle between baseline and follow-up or to report recurrence rate per joint separately.

In conclusion, we present a uniform definition that for the first time allows comparison between future studies, thereby improve our understanding of the effectiveness of different treatment methods.
