# Correction: Recurrence of Dupuytren’s contracture: A consensus-based definition

**DOI:** 10.1371/journal.pone.0216313

**Published:** 2019-04-25

**Authors:** Hester J. Kan, Frank W. Verrijp, Steven E. R. Hovius, Christianne A. van Nieuwenhoven, Dupuytren Delphi Group, Ruud W. Selles

In our article on a consensus definition of recurrence disease in Dupuytren’s disease [1], we failed to acknowledge a similar Delphi-based study by Felici et al. [2] that was published in *Handchirurgie—Mikrochirurgie—Plastische Chirurgie*. This article, as ours, provides a much more specific and detailed description of recurrence than used previously (for review on the different definitions of recurrence used in the literature, see [3]). Both Delphi studies were developed and performed separately with a different group of experts and asking different questions during the Delphi rounds.

The consensus described in the paper by Felici et al. [2] was that recurrence should be measured the level of the individual joint with a baseline measurement at 6 weeks to 3 months postoperatively. A recurrence is then defined as a passive extension deficit increase of more than 20 degrees for at least one treated joint, in the presence of a palpable cord, compared to baseline. In our article [1], we defined recurrence as more than 20 degrees of contracture in any treated joint at one year post-treatment compared to six weeks post-treatment, with recurrence reported individually for every treated joint.

When comparing both definitions, both agree on a number of important aspects that are different from previous literature (for review, see [3]), such as focusing on the individual joint as a level of analysis and on using an increase of 20 degrees of contracture as a threshold for recurrence compared to a post-operative (and not intra-operative) baseline. What differs is that our consensus does not include the presence of palpable cords as a necessity of recurrence. In addition, while the study of Felici et al. [2] does not specify a specific time point for the follow-up measurement, our Delphi group concluded on a one-year follow-up measurement, reasoning that recurrent contracture increases over time, at least in some of the patients [4, 5]. To allow comparison over studies, our consortium therefore felt a specific time point is needed, while also acknowledging that following patients longer over time should be preferred when possible. Our article [1] also adds a specific example of how to analyze a data set to clarify some of the complexities in this.

In conclusion, we feel that both papers highlight the same importance of having a recurrence definition and independently reach a largely similar conclusion except for the time-point of follow up. Both definitions should assist the field in creating better comparison of outcome studies.
